# Corneal perforation as a primary manifestation of keratoconus in a patient with underlying rheumatoid arthritis

**DOI:** 10.1007/s12348-011-0048-4

**Published:** 2011-11-17

**Authors:** Konstantinos T. Tsaousis, Nikolaos Chalvatzis, Chrysanthos Symeonidis, Nikolaos Kopsachilis, Asimina Mataftsi, Stavros A. Dimitrakos, Ioannis T. Tsinopoulos

**Affiliations:** 2nd Department of Ophthalmology, Medical School, Aristotle University of Thessaloniki, Papageorgiou General Hospital, 56429 Thessaloniki, Greece

**Keywords:** Amniotic membrane, Corneal perforation, Keratoconus, Rheumatoid arthritis

## Abstract

**Purpose:**

The purpose of this study is to report a case of corneal perforation in a patient with undiagnosed keratoconus and underlying rheumatoid arthritis.

**Methods:**

This is a retrospective case study based on the patient’s medical records and followed by a brief review of the literature.

**Results:**

A 53-year-old patient was referred to our department for acute pain and sudden decrease of visual acuity in his left eye. Corneal perforation was detected and attributed to a previously undiagnosed and untreated keratoconus. Additional laboratory work-up and clinical examination revealed a coexisting rheumatoid arthritis. Amniotic membrane was originally transplanted in order to maintain the structural integrity and promote healing of the perforated eye. Subsequently, the patient underwent a penetrating keratoplasty, though with unfavorable results due to postoperative endophthalmitis.

**Conclusions:**

Rheumatoid arthritis may be associated with higher risk of corneal perforation in patients with ectatic degenerative diseases such as keratoconus.

## Introduction

Rheumatoid arthritis (RA) and other connective tissue disorders are frequently associated with certain types of corneal or/and scleral pathology such as peripheral ulcerative keratitis, dry eye syndrome, and scleritis [1]. It is also known that these conditions may lead to perforation of the eye by compromising the integrity of its outermost layers.

Keratoconus (KC) is generally regarded as an isolated condition with no apparent ophthalmic or systemic associations at the time of diagnosis [2]. However, elevated levels of corneal proinflammatory cytokines such as interleukin (IL)-1 have been linked to corneal thinning, implying the involvement of the immune system in the pathogenesis of KC [3]. Only a small number of autoimmune diseases (e.g., RA, asthma, inflammatory bowel disease) have been related to KC, while others have not (e.g., systemic lupus, Crohn’s disease, myasthenia gravis, and multiple sclerosis) [4].

Cell-mediated mechanisms are suggested to play a pivotal role in the pathogenesis of ectatic corneal disorders and KC itself [5]. Further, confocal microscopy has revealed that in patients with RA, the central corneal and stromal thicknesses are significantly lower compared with controls, and these patients have significantly higher numbers of hyperreflective stromal cells [6]. The activation of these keratocytes is mediated by proinflammatory cytokines, such as IL-1 and IL-6 [7]. We report a case of paracentral corneal perforation in a patient with keratoconus and a previously undiagnosed connective tissue disorder.

## Case report

A 53-year-old man presented in the emergency room complaining of severe ocular pain with sudden onset and a significant decrease in visual acuity in his left eye. Regarding his ocular history, the patient noted that his ophthalmologist had been modifying his spectacle prescription annually over the last 13 years because of the increasing myopic shift. The patient reported no systemic drug intake except for analgesics for constant lumbar back pain. His systemic history was otherwise unremarkable.

On ophthalmic examination, the patient’s best corrected visual acuity was measured CF at 10 cm for the left and 20/60 for his right eye. His spectacle prescription at the time of the examination was: −1.00/−5.5 × 100° OS and −4.5/−4.5 × 70° OD.

**Fig. 1:**
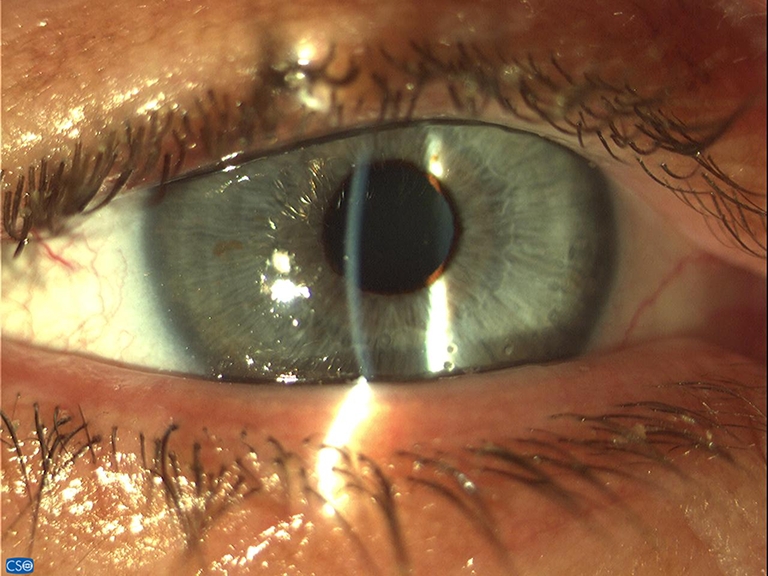
Photo of the fellow eye. Steep corneal curvature on biomicroscopy raised suspicions of a coexistent keratoconus

**Fig. 2:**
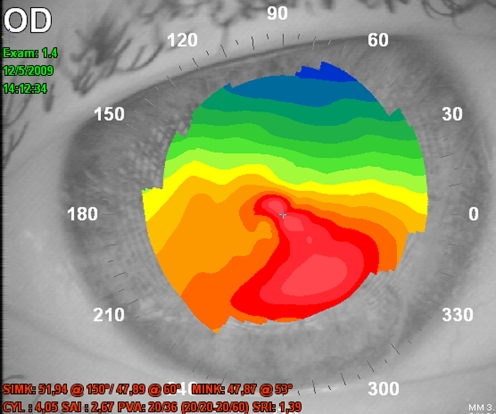
Topography image demonstrating significant keratoconus on the right eye

Slit lamp biomicroscopy revealed a paracentral corneal perforation, 1 × 2 mm, accompanied by iris prolapse, corneal thinning, and a markedly shallow anterior chamber in the left eye. The right eye displayed clinical features of moderate keratoconus, which was confirmed by corneal topography (Magellan Mapper, Nidek; Figs. 1 and 2).

**Fig. 3:**
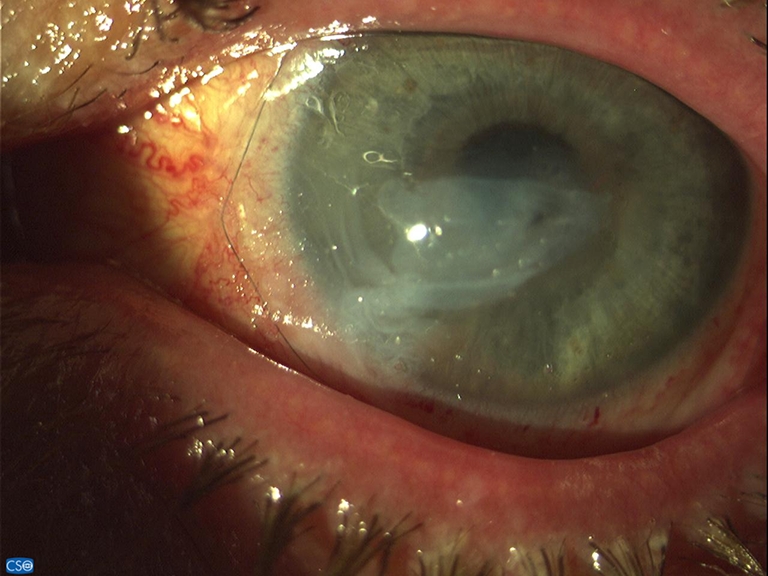
Photo of left eye on second post-op day after amniotic membrane transplantation in multiple layers in an attempt to maintain the eye’s anatomical integrity

The patient was immediately prepared for surgical intervention in order to preserve the integrity of the affected eye [8]. Amniotic membrane (AM) was primarily available and transplanted in an attempt to seal the perforation and promote corneal healing (Fig. 3). Cryopreserved AM was attached over the perforation in multiple layers and covered with an epithelial-side-up, limbal-sutured sheet. The surgical technique involved scraping of residual corneal epithelium and 360° limbal peritomy of the conjunctiva prior to AM transplantation. The outermost of the multiple layers was sutured with 8/0 vicryl on the cornea whereas the overlaid transplant was sutured at the limbus with a running 10/0 nylon. A bandage contact lens was applied at the end of the operation in order to promote reepithelialization of AM and to prevent mechanical rubbing of the transplant on blinking.

Further blood tests revealed a significantly elevated rheumatoid factor at 2,170 IU/ml (reference value <20 IU/ml). Specific blood tests in addition to detailed clinical examination carried out by an orthopedic surgeon and immunologist confirmed the diagnosis of RA, and the patient was referred to rheumatologists for further assessment.

The early postoperative results following AM transplantation were encouraging based on ophthalmic examination. The operated on eye displayed a well-formed anterior chamber, without any leakage, over the 3 post-op weeks. Visual acuity was slightly improved to CF at 50 cm. However, reepithelialization failed to cover AM completely whilst subsequent tissue thinning resulted in re-leakage at week 4 postoperatively. The condition deteriorated further over the following weeks, necessitating an urgent penetrating keratoplasty. One month after corneal grafting and while on systemic immunosuppressants, the patient developed endophthalmitis with unfavorable outcome. He eventually underwent evisceration of his eye and was lost to follow-ups.

## Discussion

KC is a noninflammatory corneal disorder in which stromal thinning and ectasia result in an atypical corneal shape. Although the etiology is not completely understood, there is evidence suggesting that KC probably represents a multifactorial process. KC with acute hydrops can be complicated with spontaneous corneal perforation; however, this has been reported mainly in cases with advanced KC. Nevertheless, spontaneous perforation in mild to moderate KC cases has also been reported recently [9].

Ophthalmic involvement in autoimmune disorders such as RA, systemic lupus erythematosus, and Sjögren syndrome has been extensively described. However, many patients experience ocular sequelae during the course of undiagnosed immunological disorders. Reduced tear secretion, chronic inflammation of the ocular surface, as well as ultrastructural abnormalities are believed to play a major part in decompensating the ocular surface. This condition may lead to spontaneous perforation in sporadic cases. In our case, AM transplantation was used as a first-line solution since previous studies showed that urgent penetrating keratoplasties in perforated eyes displayed higher rates of graft failure compared to those performed several weeks to months following the perforation [10, 11].

Multilayer AM transplantation has been reported to promote epithelial healing and reduce inflammation, neovascularization, and scarring in a diversity of external eye disorders [12]. AM-related local anti-inflammatory effects contribute to faster and more efficient reepithelialization. Furthermore, AM serves as a biological scaffolding in cases where normal tissue is lost and promotes reinforcement or even sealing of small perforations.

AM has been used in patients with RA-related epithelial defects as a temporizing solution with the aim for delayed reconstruction. However, as Solomon and colleagues reported [8], AM transplantation showed poorer outcomes in patients with systemically undertreated RA compared to other autoimmune conditions like ocular pemphigoid or even Sjogren’s. Other authors have proposed cyanoacrylate and fibrin glue adhesives as an efficacious solution in cases where corneal perforations occur [13, 14]. In our case, AM transplantation was preferred over other treatment modalities mainly due to severe inflammation of the ocular surface and the small size of the perforation. Besides, the diagnosis of RA had not yet been established. Furthermore, penetrating keratoplasty does not represent a first-choice operation in our country due to the relatively restricted number of centers authorized to perform corneal transplantations and to limited availability of corneal grafts. In our patient, although the AM transplant initially provided a sufficient corneal sealing, it failed as a long-term solution due to poor reepithelialization and the progressive tissue degradation. Recurrent aqueous humor leak necessitated an emergent penetrating keratoplasty, which took place in another center.

There are quite a few studies that extensively describe the relationship between RA and ocular surface disorders that may permanently affect vision [15, 16]. KC is another thoroughly studied condition that, apart from major refractive alterations, may occasionally cause corneal perforation when undiagnosed or/and untreated.

## Conclusion

To the best of our knowledge, this is the first report of a case with corneal perforation arising from coexistent KC and RA. It seems possible that the impact of these conditions is more severe when they interact. We should therefore raise awareness and either rule out or efficiently treat underlying connective tissue disorders in patients with KC.
